# Novel role of COX6c in the regulation of oxidative phosphorylation and diseases

**DOI:** 10.1038/s41420-022-01130-1

**Published:** 2022-07-25

**Authors:** Changyu Wang, Jianjun Lv, Chengxu Xue, Jiawen Li, Yanqing Liu, Danni Xu, Yuting Jiang, Shuai Jiang, Minghui Zhu, Yang Yang, Shaofei Zhang

**Affiliations:** 1 Department of Cardiology, Xi’an No. 3 Hospital, The Affiliated Hospital of Northwest University, Faculty of Life Sciences and Medicine, Northwest University, Xi’an, China; 2 Key Laboratory of Resource Biology and Biotechnology in Western China, Ministry of Education, Faculty of Life Sciences, Northwest University, Xi’an, China

**Keywords:** Molecular biology, Diseases

## Abstract

Cytochrome c oxidase subunit VIc (COX6c) is one of the most important subunits of the terminal enzyme of the respiratory chain in mitochondria. Numerous studies have demonstrated that COX6c plays a critical role in the regulation of oxidative phosphorylation (OXPHOS) and energy production. The release of COX6c from the mitochondria may be a hallmark of the intrinsic apoptosis pathway. Moreover, The changes in COX6c expression are widespread in a variety of diseases and can be chosen as a potential biomarker for diagnosis and treatment. In light of its exclusive effects, we present the elaborate roles that COX6c plays in various diseases. In this review, we first introduced basic knowledge regarding COX6c and its functions in the OXPHOS and apoptosis pathways. Subsequently, we described the regulation of COX6c expression and activity in both positive and negative ways. Furthermore, we summarized the elaborate roles that COX6c plays in various diseases, including cardiovascular disease, kidney disease, brain injury, skeletal muscle injury, and tumors. This review highlights recent advances and provides a comprehensive summary of COX6c in the regulation of OXPHOS in multiple diseases and may be helpful for drug design and the prediction, diagnosis, treatment, and prognosis of diseases.

## Facts

- COX6c is one of the most important subunits of the terminal enzyme of the respiratory chain in mitochondria.
- COX6c is closely related to a variety of diseases and can be used as a potential biomarker for diagnosis and treatment.
- COX6c plays a pivotal role in the regulation of the OXPHOS pathway. Mitochondrial biogenesis can be stimulated in response to low temperature and is reflected in increases in the expression and activity of COX6c.

## Open questions

- What extent are the activity and expression of COX6c altered in various diseases?
- Is the change in COX6c a secondary event associated with other pathways that might be the primary drivers of the functional outcomes or is it specific to the disease?
- What are the specific mechanisms and processes that COX6c participates in during the development of disease?

## Introduction

The mitochondrial respiratory chain, also known as the electron transport chain (ETC), is crucial to life. Cytochrome c oxidase (COX), the terminal enzyme of the respiratory chain in the mitochondrial inner membrane, has been acknowledged as a rate-limiting step of energy production in mitochondria. It not only catalyzes the electron transfer from reduced cytochrome c to molecular oxygen but also translocates protons across the mitochondrial membrane. The proton motive force generated by COX (Complex IV), along with the other two proton-pumping enzymes, namely, Complexes I (NADH: ubiquinone oxidoreductase) and III (cytochrome bc1), subsequently drives ATP synthase and facilitates energy production through oxidative phosphorylation (OXPHOS) [1]. In humans, COX biogenesis involves the coordinated assembly of 13 subunits, the largest 3 of which, I, II, and III, are catalytic subunits encoded by mitochondrial DNA (mtDNA) and synthesized within the mitochondria. The remaining 10 structural subunits are encoded by nuclear DNA and synthesized on cytosolic ribosomes, subsequently being translocated into the mitochondrial inner membrane mainly through mitochondrial targeting and import [2–4]. The mitochondrial-encoded subunits act in electron transfer, whereas the nuclear-encoded subunits appear to be involved in the structural integrity, regulation, and dimerization of the enzyme. The presence of isoforms may reflect the different energy requirements that depend on tissues, developmental stages, or growth conditions [5, 6]. For instance, COX4-2 is the lung-specific isoform, COX6B and COX8-3 are the two testis-specific isoforms, and COX6A, COX7A, and COX8A are the three liver/heart-type subunits. In addition, heart isoforms are expressed in tissues that possess a high aerobic capacity and an abundance of mitochondria, whereas liver isoforms are found in tissues that involve fewer mitochondria, such as the brain, liver, and kidney [7]. In particular, the nuclear gene that encodes subunit VIc, COX6c, has been shown to be the most altered in a variety of diseases.

COX6c is a 933-bp linear DNA located at human chromosome 8q22.2 that comprises 4 exons and 3 introns [8, 9]. Among all these COX subunits, COX6c has no paralogs in any vertebrate lineage, including mammals. COX6c polymorphisms in exons 2 and 3 form a haplotype [10]. The mature COX6c protein is 75 amino acids long and shows 69% homology between rats and humans as well as 73% between bovines and humans [11]. However, the structure in mice requires further elucidation. In addition, the mature protein-coding region of the *COX6c* gene has been shown to undergo accelerated evolution in amino acid replacement in the human lineage, suggesting an adaptive positive selection of functional significance [12]. Although mature COX6c protein possesses a property of the presequence, it does not have a cleavable presequence that targets mitochondria and has only two amino acids cleaved from the mature protein [3]. COX6c subunits can be assembled into subcomplexes in vitro, which may represent rate-limiting intermediates, according to in vitro introduction and assembly experiments [13]. Furthermore, ubiquitous COX6c has been reported to be constitutively expressed in almost all tissues, especially the heart and colon [14].

COX6c plays a pivotal role in the regulation of the OXPHOS pathway. Mitochondrial biogenesis can be stimulated in response to low temperature and is reflected in increases in the expression and activity of COX6c [15]. It has been well established that human sperm requires mitochondrial ATP through OXPHOS [16, 17]. Amaral and colleagues [18] noted that good-quality sperm expresses a significant amount of ETC proteins, including COXI and COX6c, compared with low-quality samples. The positive correlation between the expression of COX6c and sperm quality is more important for sperm concentration and motility than morphology. Notably, there is no correlation between the percentage of sperm expressing COX6c and mtDNA copy number. This result further confirms that other factors affect COX6c expression, such as nuclear respiratory factors (NRFs), which will be discussed below.

## Novel function of COX6c beyond OXPHOS

**Fig. 1:**
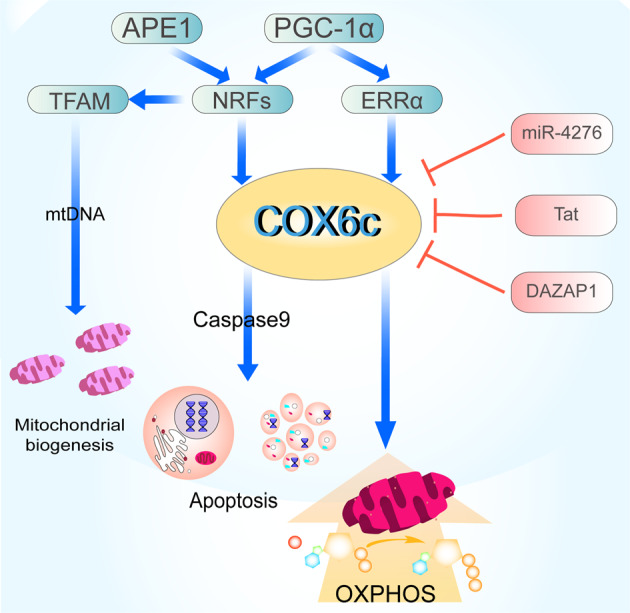
COX6c regulation and function. COX6c is regulated by multiple factors. (1) On the one hand, APE1 promotes the binding of NRFs to TFAM and regulates mitochondrial biogenesis. In addition, the expression of COX6c is regulated by the redox-dependent coactivator NRFs of APE1; (2) On the other hand, PGC-1α binds to NRFs and ERRα, respectively, to promote the expression of COX6c, which in turn promotes apoptosis and oxidative phosphorylation. During the whole process, COX6c is negatively regulated by miR-4276, Tat, DAZAP1.

The release of COX6c from mitochondria may be a telling sign of the intrinsic apoptotic pathway, which is consistent with the significant function that COX6c plays in modulating OXPHOS (Fig. 1). Interleukin (IL)-24 controls the production of several genes involved in cell proliferation and death, according to research by Hadife and colleagues [19]. Within the first 6 h, the transcription of genes related to DNA replication and metabolism, such as cell division cycle 6 (CDC6), is inhibited in the B-cell differentiation model. In the later 36 h, IL-24 stimulates COX6c expression and triggers the mitochondrial apoptotic pathway. IL-24-mediated apoptosis in B-cells has been shown to be dependent on p53. Several lines of evidence suggest that p53 can cripple the Warburg effect, where cancer cells exhibit a preferential utilization of glycolysis over aerobic respiration to produce ATP. The related mechanisms involve the regulation of the expression of synthesis of COX (SCO2), a copper-binding protein that is essential for the assembly of COX [20, 21]. Moreover, COX6c expression is remarkably suppressed by glucose, which is also supported by evidence that cells grown in the presence of lactate exhibit approximately 5 times higher COX6c mRNA levels than those grown in glucose [22, 23]. It will be of interest to determine whether COX6c expression is further boosted by p53 under stress conditions, such as glucose deprivation.

Othumpangat and colleagues [24] observed that during the first 3 h after the influenza virus exposure, host cells boost COX6c mRNA expression by silencing miR-4276. Moreover, COX6c in turn inhibits viral replication via activation of caspase-9. This indicates an initial cellular defense that eliminates infected cells by driving them into an apoptotic state. Noticeably, when the virus takes control of the cell, miR-4276 induction represses the expression of COX6c and caspase-9, allowing the cell to transform into an antiapoptotic state and finally promoting viral replication and spread. Since the expression of miR-4276 and COX6c is not specific to the viral strain and there is an antigenic shift and drift of the virus, miRNA inhibitors may be more broadly applicable than vaccines that target a specific virus. Thus, these data strongly render a more effective approach to antiviral therapy.

## Regulation of COX6c

Peroxisome proliferator-activated receptor γ (PPARγ) coactivator 1 (PGC-1α), the energetic regulator, is acknowledged as a critical signaling pathway regulating COX6c expression [25]. As a powerful regulator of cellular energy metabolism, PGC-1α is primarily expressed in tissues and organs rich in mitochondria, such as the heart, liver, kidney, brain, brown adipose tissue, and skeletal muscle. PGC-1α initiates the expression of a wide range of coactivated genes involved in virtually all aspects of mitochondrial energy metabolism [26]. NRF-1, one of the most important coactivated targets of PGC-1α, remarkably activates the expression of the gene that encodes COX6c through binding to its promoter [27, 28]. Moreover, NRF-1-binding sites are also identified in the promoter of mitochondrial transcription factor A (TFAM), which subsequently binds to mtDNA and exerts significant effects on mtDNA replication, transcription, and maintenance [26]. The transcriptional activity of both NRF-1 downstream target genes, COX6c and TFAM, is enhanced by an increase in NRF-1 activity upon hydrogen peroxide-induced oxidative stress [29, 30]. The other isoform, NRF-2, plays a parallel role in the transcriptional regulation of COX6c [31, 32]. Furthermore, the human apurinic/apyrimidinic (AP) endonuclease 1 (APE1) has been demonstrated to play an additional regulatory part in the DNA-binding and transcriptional activity of NRF-1 through its redox function, further modulating mitochondrial function after oxidative stress. Knockdown or redox mutation of APE1 impairs NRF-1 DNA-binding activity, consequently reducing the expression of COX6c [29]. Hence, these results suggest that APE1 could be either the redox-dependent coactivator of NRF-1 or the redox-dependent mediator between PGC-1α and NRF-1, which needs to be further clarified. (Fig. 1)

The expression of COX6c is also induced by the estrogen-associated receptor, another PGC-1 partner, and ERRα target genes have a conserved ERR responder (ERRE) in their promoter [33, 34]. In addition, the binding of ERRα to ERRE in its own promoter stimulates its transcription in a positive autoregulatory loop once activated by PGC-1α [26]. COX6c can be used as an endpoint of ERRα activity in contracting cardiomyocytes by measuring changes in the corresponding mRNA level in response to hypoxia. As shown by Cunningham and colleagues [33], contracting adult cardiomyocytes’ adaptive response to persistent hypoxia includes the stimulation of the PGC-1/ERR/COX6c-signaling pathway. Together, the PGC-1α-signaling pathway is essential for the transcriptional regulation of COX6c and further helps maintain mitochondrial function.

COX6c activity and expression can also be negatively modulated. The human immunodeficiency virus type 1 (HIV-1) transactivator of transcription (Tat) protein is an important contributor to the HIV-induced pathogenesis of acquired immunodeficiency syndrome (AIDS), including the apoptosis of various cell types [35]. Tat protein is able to suppress COX activity in homogenates from various human tissues and organs, including the liver, heart, brain, and skeletal muscle [36]. This increases the potential of the viral protein as a COX6c inhibitor. The transcriptional activity of COX6c can be regulated at the posttranslational level by miRNAs, such as miR-4276, which has been discussed above. Moreover, modifications of COX6c expression include a decrease in COX6c pre-mRNA splicing efficiency by DAZAP1, which is an evolutionarily conserved RNA-binding protein that can regulate gene transcription and translation [37, 38]. This is demonstrated by the overexpression of DAZAP1, which directly contributes to the accumulation of unspliced COX6c pre-mRNA, further inhibiting COX6c. It is notable that DAZAP1 loads onto COX6c mRNA with just the last intron of the COX6c gene present, indicating the occurrence of specific intron-dependent mRNA binding. In addition, DAZAP1 knockdown causes the overexpression of COX6c, which ultimately slows the proliferation of HEK293 cells [9]. Overall, DAZAP1 is identified as a negative regulator of COX6c expression and may control energy production in mitochondria.

## COX6c and diseases

COX6c plays a key part in endocrine, urinary, reproductive, circulatory, respiratory, and other systems. This section mainly introduces its relationship with diseases of different systems, such as cardiovascular disease, and diabetic nephropathy (DN).

### COX6c and cardiovascular disease

Cardiovascular diseases such as atherosclerosis are common diseases of the human circulatory system, which affect the life quality of human beings. Atherosclerosis is an inflammatory progressive disease attributed to the deposition of lipids, namely, cholesterol and cholesterol esters, to the intima of the arterial wall. This vascular disease is associated with various risk factors, such as hypercholesterolemia, hypertension, and diabetes [39]. The analysis of KEGG signaling revealed significant enrichment of differentially expressed genes, including the COX6c gene in the OXPHOS pathway in familial hypercholesterolemia patients [40]. Since mitochondria are also a prime cellular source of reactive oxygen species (ROS), the underlying mechanism may entail the ROS accumulation in COX6c, which contributes to the increase in oxidative stress, ultimately hastening the evolution of hypercholesterolemia and atherosclerosis [40, 41]. Moreover, the downregulation of COX6c expression in blood samples from familial hypercholesterolemia patients suggests that aberrant expression of COX6c may result in the development of atherosclerosis through the OXPHOS pathway [40]. These data strongly indicate that the *COX6c* gene may function as a potential marker for the prediction and treatment of atherosclerosis (Table 1).

**Table 1:** Significance of COX6c in various diseases.

Disease	Model	COX6c	Significance	Reference
Cardiovascular	Familial hypercholesterolemia	Differentially expressed gene (↓)	Prediction of genetic risk factors of atherosclerosis; A potential therapeutic target	[40]
Kidney	Diabetic nephropathy	Differentially expressed gene (↑)	Telmisartan can downregulate COX6c and improve kidney function of diabetic	[44, 45]
	ESRD/HD	Expression ↓	A promising therapeutic target	[25]
	ESRD/HD	Expression ↓	A negative correlation with oxidative stress	[28]
	ESRD/PD	Expression ↓	An indicator of mitochondrial oxidative metabolism	[48]
Brain	Alzheimer’s disease	Expression ↓ Activity ↓	Secondary event in brain areas associated with impaired metabolic activity and neuronal loss	[10, 50]
	Ischemia	Expression ↑	Compensatory effect to restore OXPHOS	[51]
Skeletal muscle	Contusion	Expression changes in a regular way	Diagnosis reliability in wound age estimation	[52, 53]
	Acute COPD exacerbation	Expression ↓	Downregulation of OXPHOS pathway; Upregulation of apoptosis pathway	[55]
Tumor	Prostate cancer	Expression ↑	A useful marker to study the alteration of energy metabolism in cancer cells; Diagnosis	[56]
	Drug resistance in breast cancer	Expression ↑	A compensating mechanism to maintain mitochondrial function to serve as a survival mechanism to overcome drug resistance	[60]
	Breast cancer	Expression ↓	Striking toxicity and acceleration of cell death	[62, 63]
	Breast cancer	Expression ↓	Discriminating hormone-responsive breast cancer Predicting patient survival	[65]
	Follicular thyroid cancer	COX6c/DERL2 translocation	Tumor diagnostics	[66]
	Uterine myomas	COX6c/HMGIC translocation	Tumor diagnostics	[67, 68]
	Retroperitoneal lipoma	Chromosome arrangement	Tumor diagnostics	[69]

### COX6c and kidney disease

Diabetes is a complex polygenic disease with increasing global prevalence. COX6c has been proven to use as a significant gene in both fulminant type 1 diabetes [42] and type 2 diabetes mellitus [43] to provide new therapeutic targets. It is closely related that DN is a serious endocrine system disease, which has become the single most common cause of end-stage renal disease (ESRD) in adults. Approximately 30% of patients with diabetes mellitus develop DN. Zhang et al. [44] discovered that the expression of COX6c is noticeably elevated in the glomeruli of DN rats, implying that the OXPHOS pathway may be vital to the emergence of DN. By artificially deflating COX6c expression and blocking the OXPHOS pathway, they proved that telmisartan ultimately improves renal function in diabetic rats [45]. Additionally, through PPAR, a crucial coactivated partner of PGC-1, telmisartan moderates renal function [45, 46]. Telmisartan has also been shown to down-regulate TGF-1-induced expression of α-smooth muscle actin and collagen IV in mesangial cells by activating PPAR [45]. Furthermore, telmisartan also inhibits angiotensin II receptor (AT-1R) expression and drives the expression of ROS detoxifying enzymes, such as superoxide dismutase (SOD), through the PGC-1α/PPARγ pathway, further improving kidney function together with the OXPHOS pathway [26, 46, 47].

ESRD patients need renal replacement therapies (RRTs), including hemodialysis (HD) and peritoneal dialysis (PD), to ensure their survival. It has been demonstrated that the expression of COX6c and its upstream gene PGC-1α is significantly downregulated in ESRD patients on HD therapy (ESRD/HD), indicating a reduction in OXPHOS activity [25]. Notably, the downregulation of PGC-1α is independently associated with the development of cardiovascular disease in HD patients. The pharmacological modulation of PGC-1α regulates its downstream effectors, including COX6c, NRF-1, and TFAM, and might be a promising therapeutic tool to reduce oxidative stress-related clinical complications in ESRD/HD patients. Hashad and colleagues [28] confirmed that the expression of NRF-1 and COX6c is downregulated in ESRD/HD patients. Moreover, there is a negative correlation between oxidative stress and the expression of both the NRF-1 and *COX6c* genes, and COX6c appears more relevant. In addition, Zaza and colleagues [48] found that the expression levels of PGC-1α, NRF-1, and their downstream target gene *COX6c* are significantly downregulated in ESRD/PD patients in association with increased oxidative stress. On the other hand, nuclear factor erythroid 2-related factor 2 (Nrf2), a transcription factor that induces the expression of the antioxidant enzyme SOD2, is upregulated in ESRD/PD patients [48, 49]. SOD2 then binds to the superoxide byproducts of OXPHOS and converts them to hydrogen peroxide and diatomic oxygen [48]. Together, these data strongly suggest that the decrease in mitochondrial OXPHOS activity reduces ROS accumulation and creates antioxidant feedback, and COX6c and its upstream regulators might be indicators of mitochondrial oxidative metabolism.

### COX6c and brain disorder

Decreased COX6c expression and activity have been observed in temporal and parietal cortices in patients with Alzheimer’s disease [10, 50]. However, it is worth noting that this decrease may not be specific to brain disorders but as a secondary event in brain areas associated with impaired metabolic activity and neuronal loss. As one of the components of Complex IV in the ETC, the suppression of COX6c due to a lack of oxygen is also a core initial process of brain ischemic injury. A new study using MALDI imaging mass spectrometry showed increased expression of COX6c in ischemic tissue compared to healthy opposite-brain areas [51]. The underlying mechanism of this compensatory effect involves an elevated requirement of mitochondrial function to restore OXPHOS after ischemia, which might be promoted by the increase of total mitochondrial mass. Therefore, COX6c might represent a potential diagnostic biomarker or target molecule for brain disorders.

### COX6c and skeletal muscle injury

Skeletal muscle injury is closely related to the motor system. Du and colleagues [52] found that the *COX6c* gene in combination with sTnI is more accurate than other genes in estimating the age of wounds following skeletal muscle contusion in rats. Sun and colleagues [53] confirmed that the COX6c expression level in contused skeletal muscle increases within 6 h after contusion when the main pathological feature includes edema and hemorrhage in myocytes without inflammatory cells or fibrous proliferation, and it decreases after 6 h when myocyte degeneration and necrosis occur. These results suggest that the level of COX6c is regular after contusion and may act as a suitable indicator for estimating wound age in combination with sTnI expression or pathological features.

Skeletal muscle function plays a central role in the survival and the prediction of mortality risk in patients with chronic obstructive pulmonary disease (COPD) [54]. COX6c is a hallmark for muscle function loss when acute COPD worsens [55]. The relevant mechanisms could involve the downregulation of the OXPHOS pathway and the upregulation of the apoptosis pathway, both of which are modulated by COX6c under conditions of excessive ROS production within the muscle, ultimately contributing to the alteration of myofilament contractile properties. Hence, the therapeutic intervention of COX6c might counteract the detrimental effects of exacerbation on these pathways and limit the loss of muscle function.

### COX6c and tumors

#### COX6c and prostate cancer

Prostate cancer is the most common form of cancer in males. In situ hybridization has shown that COX6c mRNA expression is upregulated in prostate tumor tissue and is the highest in scattered epithelial cells of prostate carcinoma. Besides that, the mRNA level of COX6c is relatively less in adjacent issues [56]. It has been found that the citrate level is extremely low in cancerous tissue compared with normal prostate tissue [57]. This could be due to the Krebs cycle, which is the mechanism used to produce energy from the metabolism of glucose and lipids, oxidizing citric acid [56]. This meets the increased energy requirement of the cancerous state for the process of malignancy, which may be supported by elevated COX6c. Together, these data indicate that the expression pattern of COX6c may serve as a useful marker to study the alteration of energy metabolism in cancer cells and help the diagnosis of prostate cancer (Table 1).

#### COX6c and breast cancer

Breast cancer, the most common cancer in females, is responsive to a wide range of chemotherapies. Mitoxantrone (MX), for instance, has attained clinical approval and is routinely used alone or in combination [58]. However, there is still a significant obstacle to the successful therapy of breast cancer due to the development of chemoresistance to multidrug resistance [59]. ATP-binding cassette subfamily G member 2 (ABCG2) is considered the major contributor to drug resistance in MCF-7/MX cells. ABCG2, as well as COX6c and ATP synthase expression, exhibite significantly higher in the MCF-7/MX cell line compared to normal MCF7 cell lines, indicating that all three of these genes increase MCF7 cell tolerance to MX [60]. The increase in COX6c expression appears to be a compensating mechanism to maintain mitochondrial function to produce a large amount of ATP to help ABCG2 pump the MX out of cells against a concentration gradient and to serve as a survival mechanism to overcome MX treatment. Taken together, these data prove that COX6c is a crucial factor in the development of drug resistance in cancer cells and may be critical for the reasonable design and use of new treatment strategies to effectively confront cancers.

Mid-region parathyroid hormone-related protein has been shown to remarkably restrain proliferation and contribute to striking toxicity and accelerate cell death in breast cancer cells [61]. The underlying molecular mechanisms may involve transcriptional reprogramming, especially downregulation of the expression of COX6c and apoptotic genes [62, 63]. Since anticancer therapeutic agents are diverse and highly targeted, there is a necessity for identifying molecularly defined subtypes of breast cancer as well as predictive and prognostic biomarkers. It has been demonstrated that COX6c plays a crucial role in the identification of hormone-responsive breast cancer or estrogen receptor (ER)+ subtypes [64, 65]. Additionally, in predicting patient survival, models incorporating COX6c protein expression together with three other proteins (GATA3, NAT1, and ESR1) significantly outperform baseline models (age, tumor size, and nuclear grade) according to a quantitative assessment of protein expression levels by Emerson and colleagues [65]. This model also offers equivalent prognostic value as lymph node status alone, but the best model is to combine the protein expression data with the nodal status. These results will help further research studies of drug responsiveness and clinical studies of patient prognosis.

#### COX6c and other tumors

COX6c helps to understand not only the etiology of prostate and breast cancers but also other cancers, including follicular thyroid cancer, uterine myomas, and retroperitoneal lipoma, which aids in tumor diagnosis. Previously, a novel COX6cC/DERL2 translocation in follicular thyroid carcinoma was detected [66]. This somatic alteration is associated with the endoplasmic reticulum and cellular metabolism. Furthermore, the new gene fusion of COX6c to HMGIC is one of the tumorigenic mechanisms in the development of uterine myomas. The first three exons of the HMGIC gene at 12q15, which encode the three DNA-binding domains, are fused to exon 2 of the COX6c gene at 8q22.2, according to nucleotide sequences of the fusion transcript [67]. The fact that Mine and colleagues [68] validated the COX6c/HMGIC translocation in uterine myomas suggested that the fusion partner COX6c gives rise to the detachment of the DNA-binding domains of HMGIC from the spacer and the acidic carboxyl-terminal regulatory domain, thereby promoting tumor growth. Moreover, COX6c has been proposed as a potential participant in the chromosomal organization in a retroperitoneal lipoma instance [69]. Yun et al. also found that COX6c is a key gene in bladder cancer tumors by detecting bladder cancer chromosomal copy number aberrations in urinary sediment [70].

Extracellular vesicles (EVs) obtained from metastatic tissue from melanoma patients have higher levels of mitochondrial membrane proteins, including MT-CO2 (encoded by the mitochondrial genome) and COX6c (encoded by the nuclear genome), than EVs isolated from non-melanoma-derived cells [71]. In gastric cancer, mRNA for LncMI-AS1 and NDUFA4 is upregulated. The expression levels of crucial indicators in the oxidative phosphorylation pathway, such as COX6C, COX5B, and NDUFA8, are observably elevated when lncMIF-AS1 or NDUFA4 are overexpressed in AGS cells [72]. Altogether, these data suggest the involvement of COX6c in gene rearrangement during tumorigenesis, providing additional tools for tumor diagnostics.

## Conclusions and perspectives

This review described the pivotal role of COX6c in various diseases. COX6c is one of the most important subunits of the terminal enzyme of the ETC in mitochondria that regulates the OXPHOS pathway and energy production. Moreover, the release of COX6c from the mitochondria may be a hallmark of the intrinsic apoptosis pathway. Notably, we emphasize the promising future of COX6c in tumor therapy and highlight the potential of COX6c in organ protection.

PGC-1α, as well as its coactivated partners, NRFs and ERRs, is essential for the transcriptional regulation of COX6c and further helps maintain mitochondrial function. COX6c activity and expression can also be negatively modulated by multiple molecules. The upregulation of COX6c expression has been determined to meet the increased energy requirement for the process of malignancy or restoration through the OXPHOS pathway. The downregulation of COX6c expression is mainly associated with decreased energy production along with oxidative stress. According to a recent study, COX6c is also a candidate biomarker for liver metabolism, which entails the addition or exposure of functional groups to the substrate through oxidation, reduction, and hydroxylation to elevate the substrate’s solubility in water [73]. There are also important involvements of COX6c in gene rearrangement during tumorigenesis and in the development of drug resistance in cancer cells. Therefore, the expression pattern of COX6c can be chosen as a useful marker of the OXPHOS pathway to study the alteration of energy metabolism and to help the prediction, diagnosis, treatment, and prognosis of various diseases.

Irradiation appears to induce a regularizing balance in cancer cell function and decrease lymph metastases. Notably, COX6c is also associated with ionizing irradiation, as shown by the considerably increased levels of COX6c found in human esophageal cancer cells following radiation exposure [74]. In another study, low-intensity laser irradiation enhanced cellular activity and boosted ATP generation in a human skin fibroblast cell type. COX6c is increased in irradiation injured and diabetic wounded cells, promoting wound repair [75]. This effect is achieved by increases in DNA, RNA, and thus protein synthesis, which is supported by an increase in ATP. Thus, COX6c modulated in the early response to irradiation may be helpful for understanding the molecular basis of radiotherapy. More work is required to elucidate the role of COX6c targeted by irradiation before developing strategies to augment its beneficial effects or establishing novel alternative adjuvant therapies.

In addition, other targeting strategies, for example, COX6c knockout or inhibitors may provide a key role in disease treatment, but there are currently no studies to support this strategy. Cyclooxygenase-2(COX-2) is also an important target in cardiovascular disease and plays a similar role to COX6c in inflammation-related diseases. Nonsteroidal anti-inflammatory drugs (NSAIDs) that are non-selective inhibitors of COX-2 inhibit inflammation while reducing angiogenesis and form a certain anti-cancer role [76]. However, when NSAIDs inhibit COX-2, they will destroy COX-1 which maintains the physiological function of cells, tissues, and organs [77, 78]. NSAIDs provide an important direction for the development of suitable inhibitors targeting COX6c for the treatment of cardiovascular diseases.

As was already established, aberrant COX6c expression is seen in various disorders, including familial hypercholesterolemia, chronic renal disease, diabetes, breast cancer, prostate cancer, uterine fibroids, follicular thyroid cancer, melanoma tissue [43, 67, 71], suggesting its potential as a treatment target. However, the lack of elucidation of COX6c and its molecular regulation mode has limited clinical treatment. In addition, conventional treatment drugs have pharmacokinetic limitations, and the efficacy is not obvious. It is thought-provoking that some studies have used nanomaterials to encapsulate drugs to target intracellular proteins to achieve the purpose of treating diseases, which has the advantages of high targeting and high circulation efficiency. For example, solid lipid nanoparticles are solid lipid-based nano-drug delivery vehicles with good physical stability and tolerance and can protect drugs from degradation and control drug release at non-targeted sites [79].

In addition, a new bioactive component—vesicle-like nanoparticles—in honey (H-VLNs) are membrane-bound nano-scale particles that contain lipids, proteins, and small-sized RNAs. H-VLNs impede the formation and activation of the nucleotide-binding domain and leucine-rich repeat-related (NLR) family, pyrin domain containing 3 (NLRP3) inflammasome, which is a crucial inflammatory signal platform in the innate immune system [80]. This study provides an important idea for the treatment of inflammation-related diseases by targeting COX6c with nano-drug delivery vehicles.

However, much of this evidence is in preclinical testing, and many problems remain unresolved. For instance, to what extent are the activity and expression of COX6c altered in various diseases? Is the change in COX6c a secondary event associated with other pathways that might be the primary drivers of the functional outcomes or is it specific to the disease? What are the specific mechanisms and processes that COX6c participates in during the development of disease? Is the expression pattern of COX6c the same in diverse tissues and cells? How do one set standards for evaluating the COX6c level to predict and diagnose disease? In conclusion, further work regarding the roles of COX6c in various diseases will promote it as a potential biomarker for the prediction, diagnosis, treatment, and prognosis of various diseases.

## Supplementary information

Co-authors’ email responses
