# Supplementary material for: Novel role of COX6c in the regulation of oxidative phosphorylation and diseases
Source: Cell Death Discov. 2022 Jul 25;8:336. doi: 10.1038/s41420-022-01130-1 (PMC9314418; doi:10.1038/s41420-022-01130-1)
Supplement: Supplementary file 1 — Co-authors’ email responses [file 41420_2022_1130_MOESM1_ESM.docx]

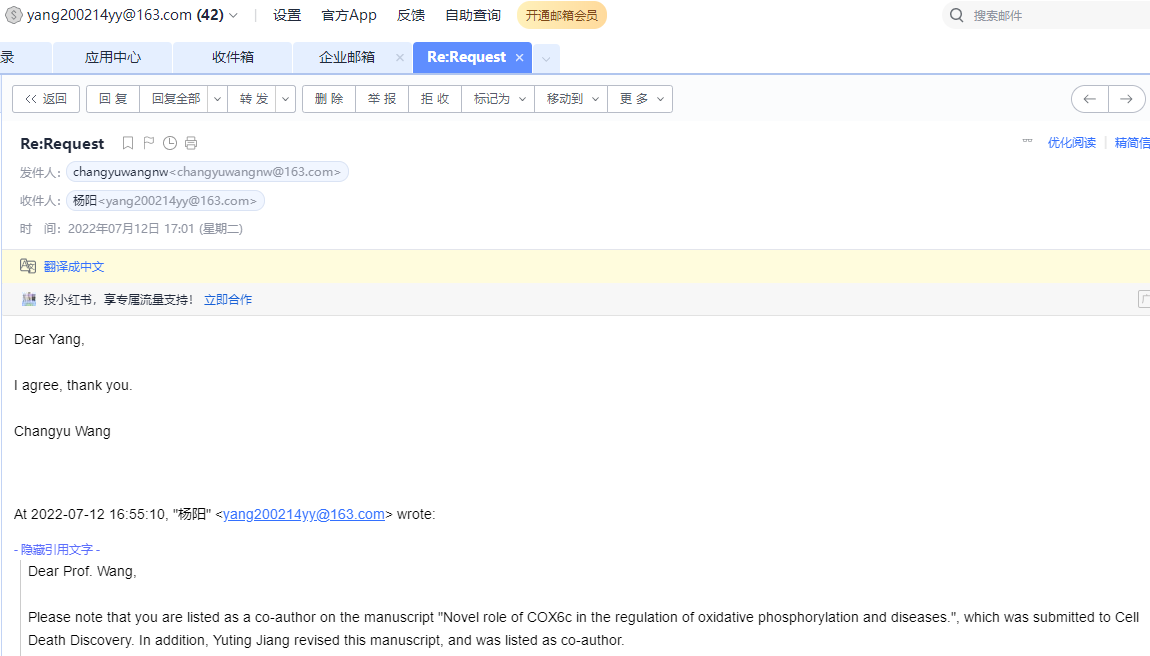


Changyu Wang


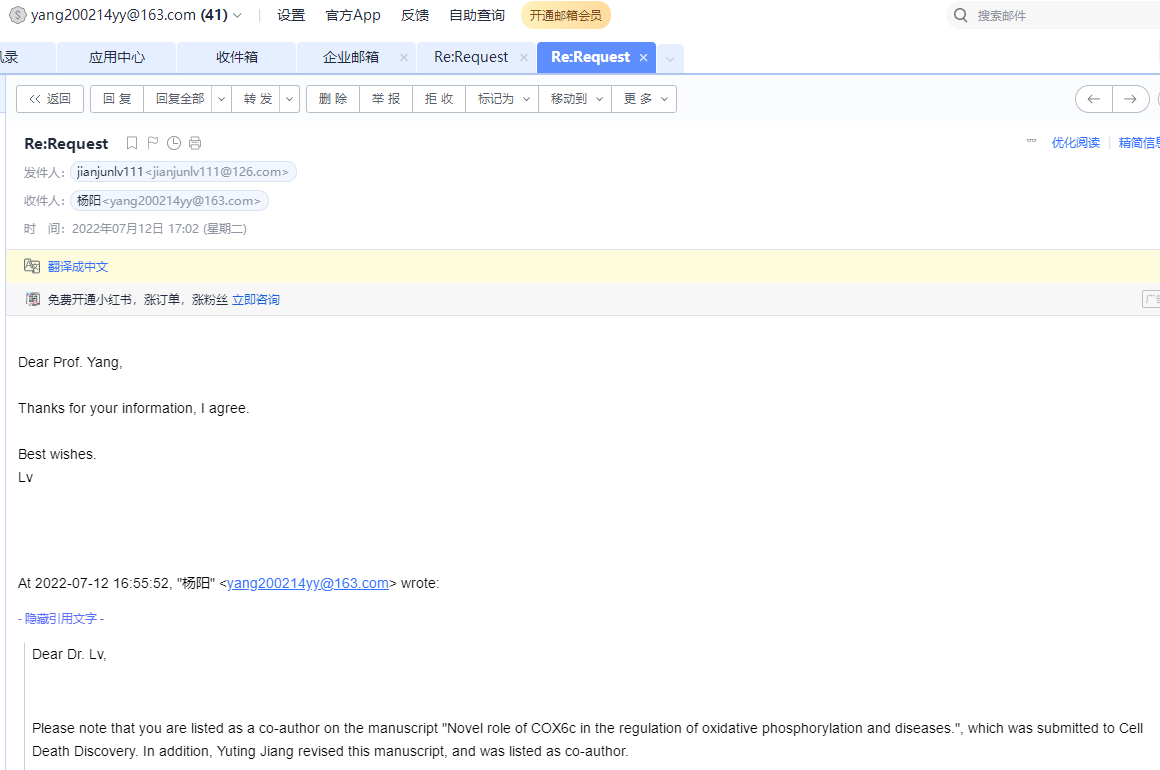


Jianjun Lv


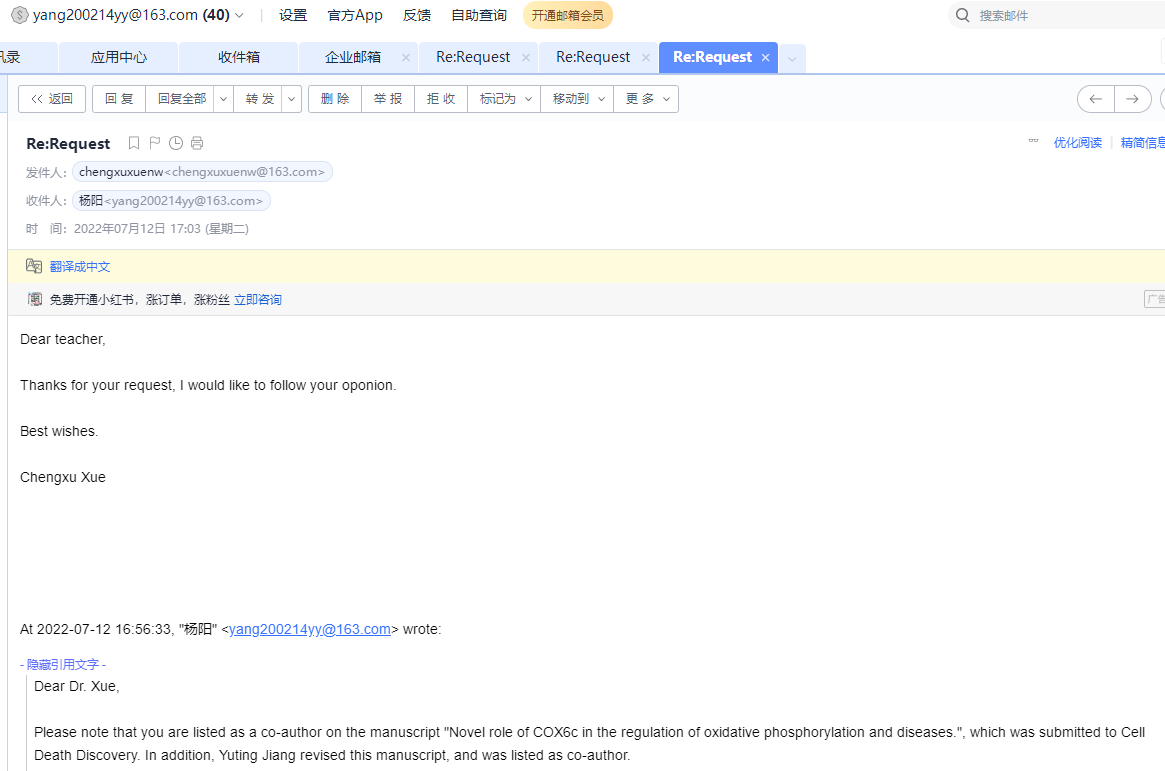


Chengxu Xue


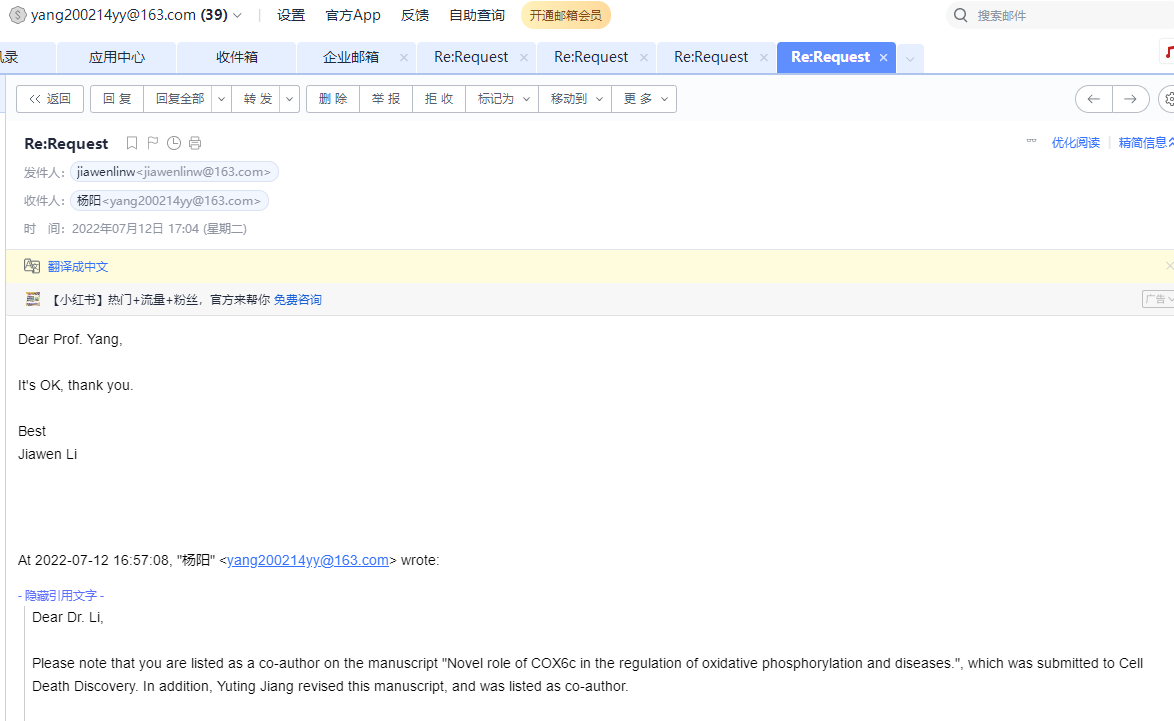


Jianwen Li


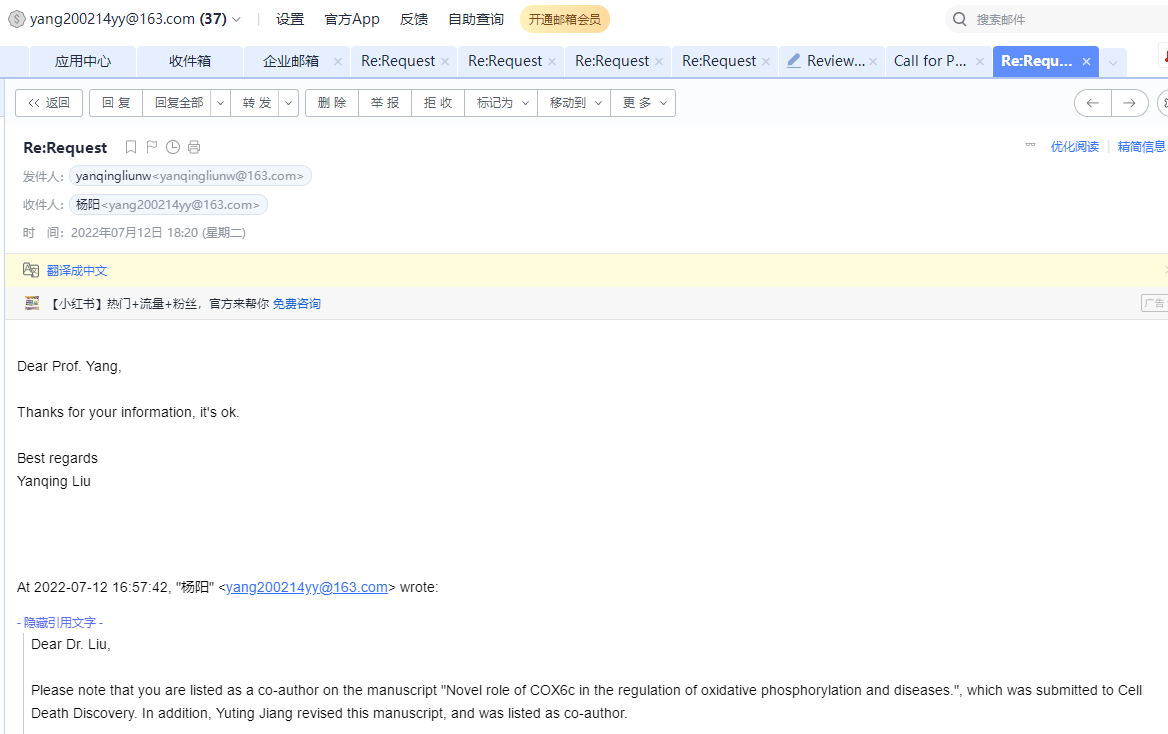

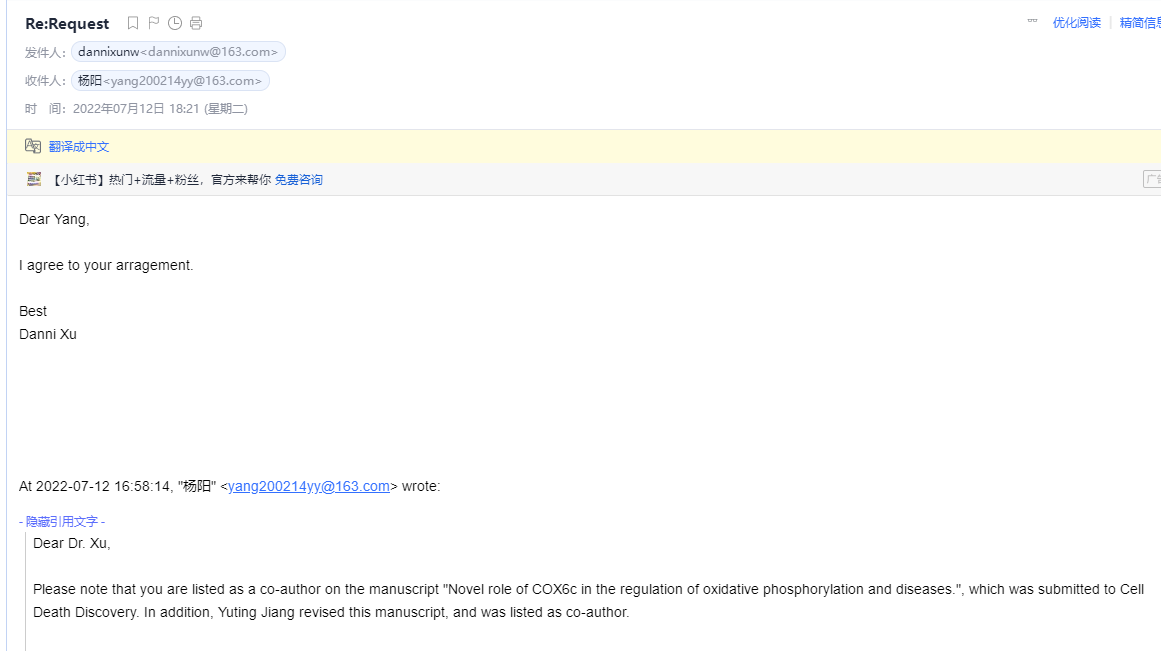


Danni Xu

Yanqing Liu


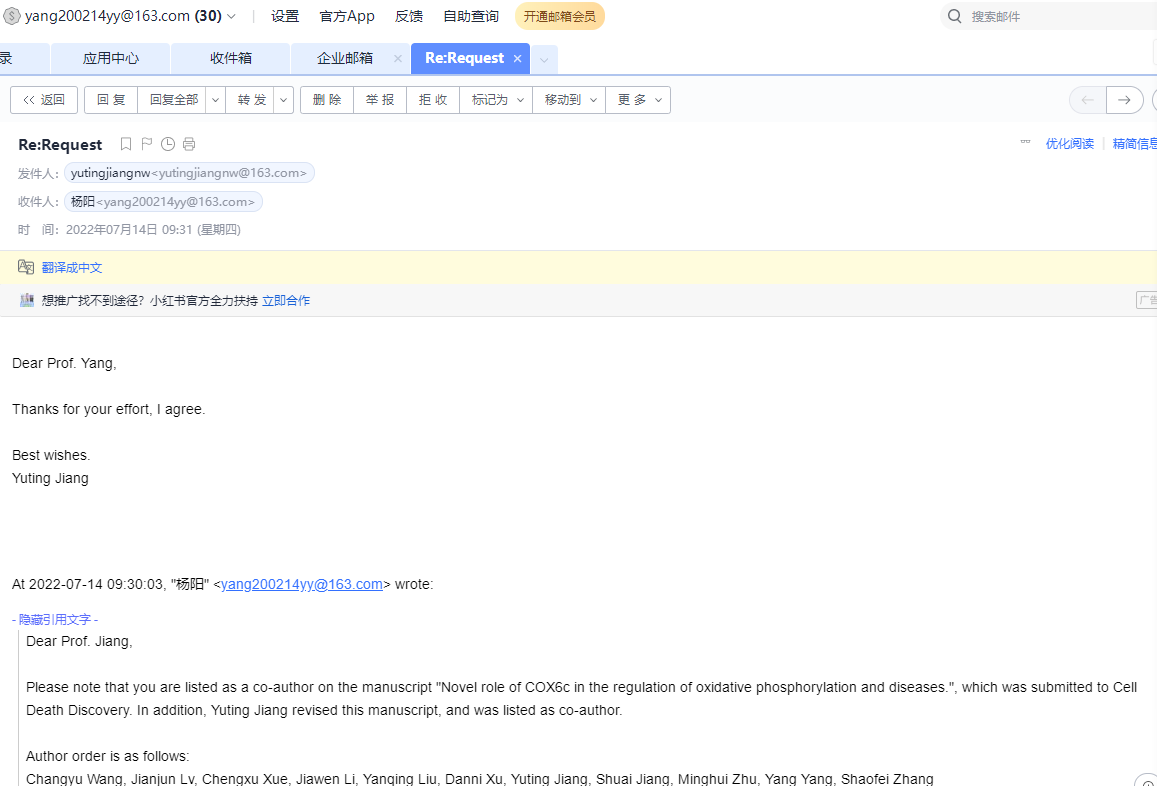


Yuting Jiang


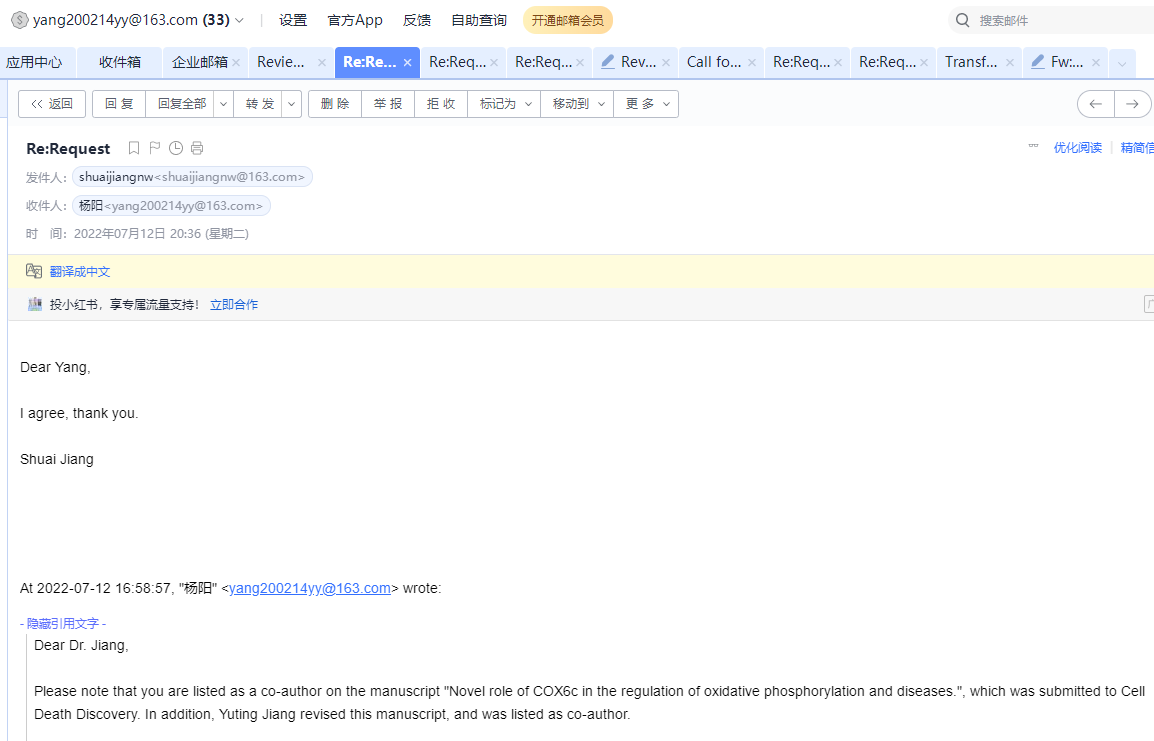

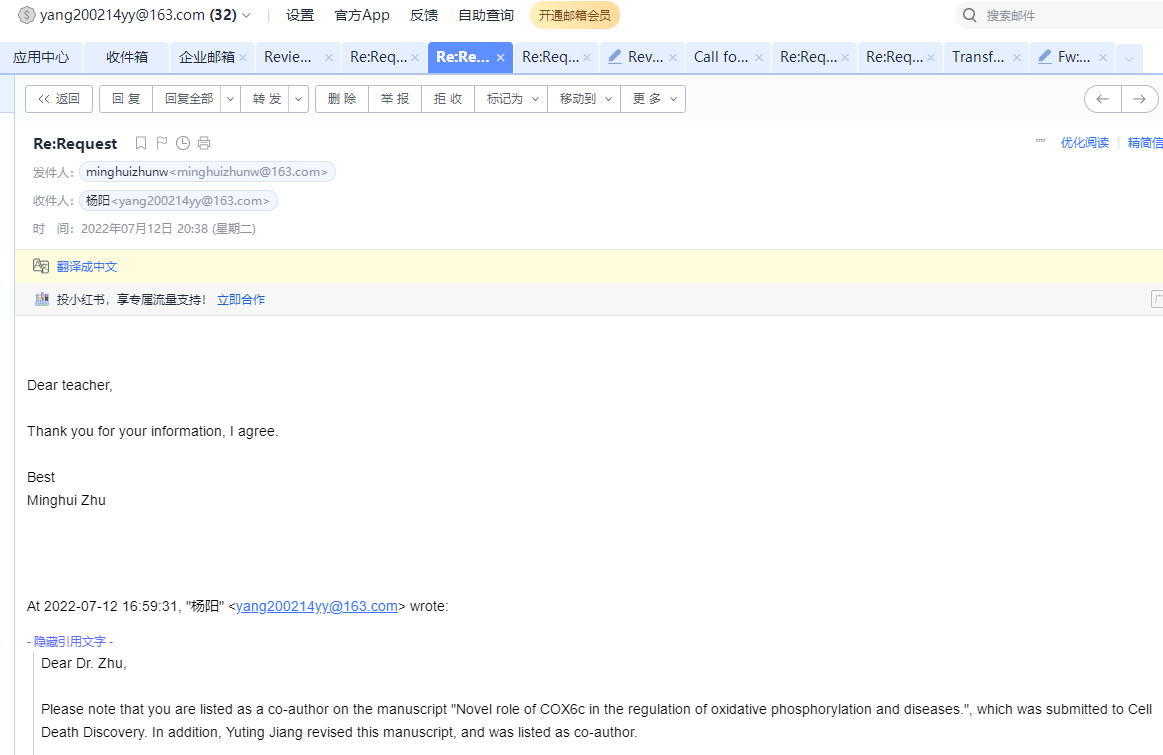

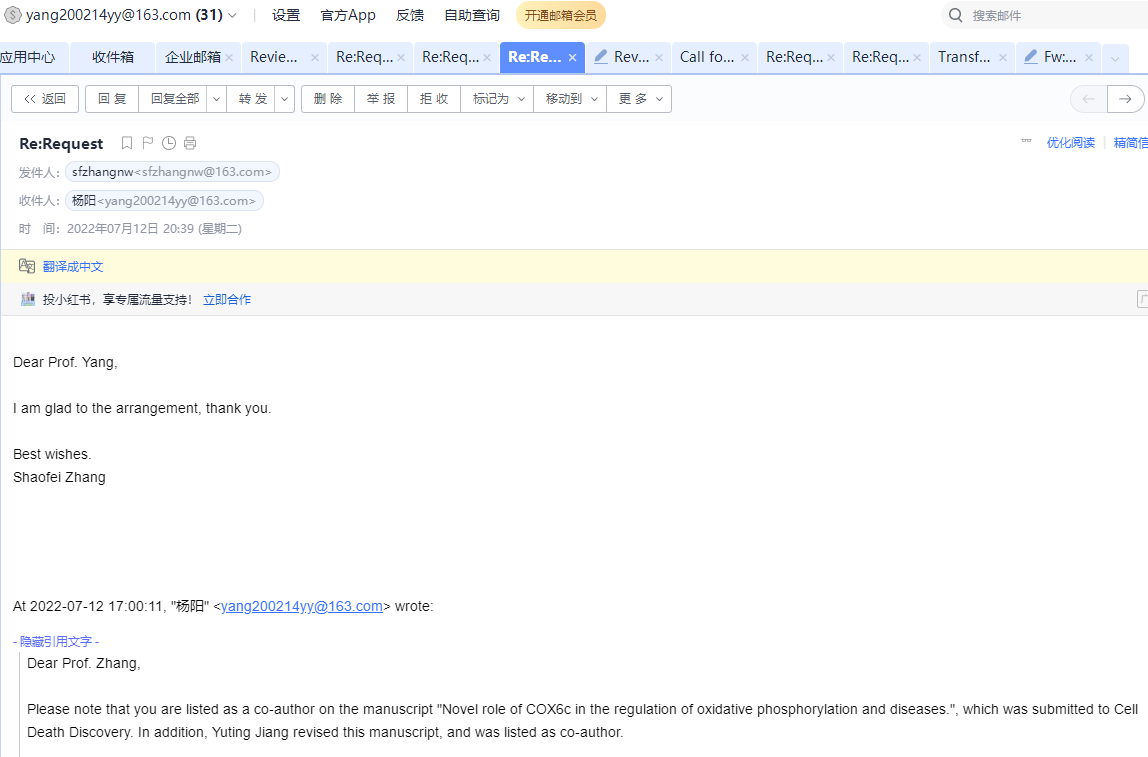


Shuai Jiang

Minghui Zhu

Shaofei Zhang


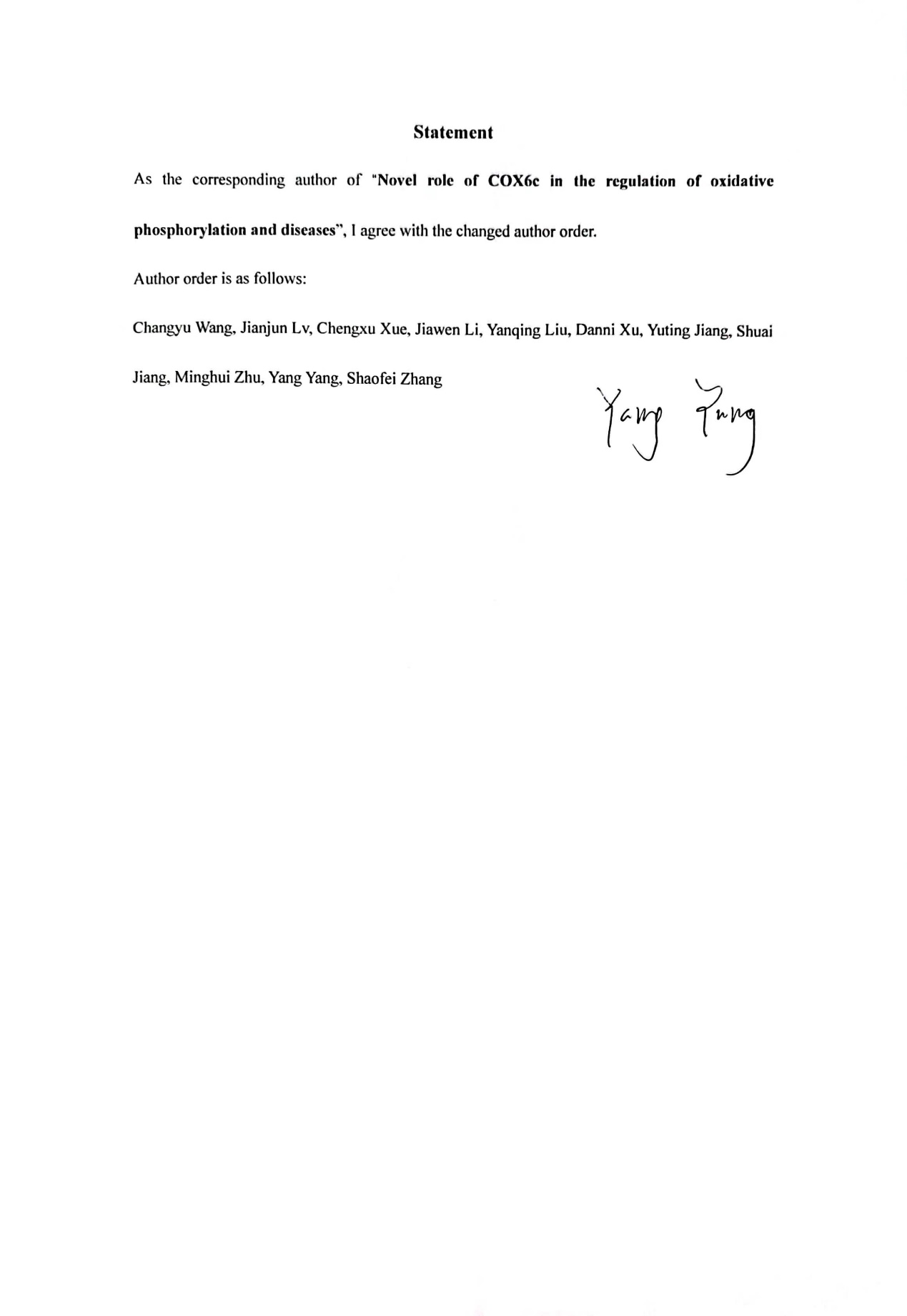


Yang Yang
